# Conceptual and Evidence Update on Incidental Physical Activity: A Scoping Review of Experimental and Observational Studies

**DOI:** 10.1111/sms.70015

**Published:** 2025-01-20

**Authors:** Daniel Reyes‐Molina, Rafael Zapata‐Lamana, Gabriela Nazar, Igor Cigarroa, Jonatan R. Ruiz, Eva Parrado, Josep‐Maria Losilla, Carlos Celis‐Morales

**Affiliations:** ^1^ Doctorado en Psicología, Facultad de Ciencias Sociales Universidad de Concepción Concepción Chile; ^2^ Escuela de Kinesiología, Facultad de Salud Universidad Santo Tomás Los Ángeles Chile; ^3^ School of Cardiovascular and Metabolic Health University of Glasgow Glasgow UK; ^4^ Escuela de Educación, Campus Los Ángeles Universidad de Concepción Concepción Chile; ^5^ Centro de Vida Saludable Universidad de Concepción Concepción Chile; ^6^ Departamento de Psicología, Facultad de Ciencias Sociales Universidad de Concepción Concepción Chile; ^7^ Escuela de Kinesiología, Facultad de Ciencias de la Salud Universidad Católica Silva Henríquez La Florida Chile; ^8^ Department of Physical Education and Sports, Faculty of Sports Science Sport and Health University Research Institute (iMUDS) Granada Spain; ^9^ Instituto de Investigación Biosanitaria de Granada (Ibs.GRANADA) Granada Spain; ^10^ Centro de Investigación Biomédica en Red: Fisiopatología de la Obesidad y Nutrición (CIBEROBN) Instituto de Salud Carlos III Madrid Spain; ^11^ Departamento de Psicología Básica, Evolutiva y de la Educación Universidad Autónoma de Barcelona Bellaterra Spain; ^12^ Instituto de Investigación del Deporte Universidad Autónoma de Barcelona Bellaterra Spain; ^13^ Department of Psychobiology and Methodology of Health Science Autonomous University of Barcelona, UAB Barcelona Spain; ^14^ Human Performance Lab, Education, Physical Activity and Health Research Unit University Católica del Maule Talca Chile; ^15^ Centro de Investigación en Medicina de Altura (CEIMA) Universidad Arturo Prat Iquique Chile

**Keywords:** behavior, definition, incidental physical activity, scope review

## Abstract

Promoting incidental physical activity (IPA) can help reduce sedentary lifestyles and physical inactivity levels in the population. However, there is heterogeneity in the definition of IPA, and studies have yet to synthesize the empirical findings on this topic. This review aimed to (1) Synthesize the definitions of the IPA used in the scientific literature, (2) Identify the behaviors part of the IPA, and (3) Synthesize the main findings on IPA. The review followed PRISMA guidelines. A systematic search was performed in July 2023, and an update was made in February 2024 in the CINAHL databases by EBSCOhost, Cochrane Library, Pubmed, ScienceDirect, Scopus, and Web of Science. The search phrase was (“incidental physical activity” OR “incidental physical activity of daily living” OR “incidental movement” OR “vigorous intermittent lifestyle physical activity” OR “VILPA” OR “physical activity of daily living”). Fifty‐five studies were included, with non‐experimental (40), experimental (12), qualitative studies (2), and mixed design (1). Ten different terms for IPA were identified, and a conceptual definition was included in 33 articles. Behaviors measured as part of the IPA were reported in 41 articles. These definitions describe unstructured, unplanned, and unintentional physical activities of daily living that are performed as a by‐product of an activity with a different primary purpose during free or occupational time and without specific fitness, sport, or recreation goals. Include light and vigorous intensities ranging from short sessions of < 1 min to prolonged ones. They include home activities, self‐care, gardening, occupation, active transportation, and walking. Furthermore, evidence on IPA suggests an association with a lower risk of all‐cause mortality. The findings of this review contribute to the updated study of IPA. Advances in data processing methods are needed to capture the diversity of behaviors and deepen the understanding of IPA.

## 1 Introduction

The latest update of the World Health Organization (WHO) guidelines on physical activity and sedentary lifestyle emphasizes that “*every movement counts*” and recommends that limiting sedentary time with physical activity of any intensity translates into health benefits [1]. In addition, there is growing evidence of the association between physical activity of any duration and improved health outcomes, including reduced all‐cause mortality [2, 3]. Thus, the recommendation that physical activity should be accumulated in segments of at least 10 continuous minutes has been modified by the message “*Any amount of physical activity is better than none, and the more, the better*” [1, 4].

In this sense, incidental physical activity (IPA) might be important in population health. This is because many everyday activities involve IPA, such as using stairs, walking for transportation, standing, shopping, gardening, housework, work‐related physical activity, playing with children, and walking pets, among others [5, 6, 7]. Furthermore, research indicates that IPA tends to have a higher adherence rate over time and appears to be easier to incorporate than structured physical exercises (e.g., going to the gym or attending guided classes), regardless of age or gender [5, 8, 9].

The Global Action Plan on Physical Activity 2018–2030 highlights the potential of reducing sedentary behaviors through promoting IPA [10]. This approach can gradually elevate physical activity levels, improving health [1]. Numerous population groups have reported a positive association between IPA and physical health and well‐being [11, 12, 13]. A recent 7‐year prospective study of 25 241 adults in the United Kingdom revealed that even 3–4 min bursts of vigorous IPA could significantly reduce the risk of all‐cause, cancer, and cardiovascular disease mortality [14].

While the body of evidence on IPA is expanding, there is still a need for more empirical studies. One potential challenge in this area is accurately conceptualizing and identifying IPA behaviors [15, 16]. Over the past decade, researchers have been refining the concept of IPA to provide methodological support to their studies. They generally agree that IPA encompasses any physical activity of daily life, whether at home, work/study, or during free time, that does not require additional time and does not have health/fitness or recreational purposes [11, 17, 18, 19, 20, 21, 22].

Although there is currently a delimited construct about what IPA is, it has limitations, such as a lack of agreement regarding the specific behaviors that IPA encompasses and whether the degree of intention with which the behaviors are performed determines if they are considered IPA. To the best of our knowledge, two theoretical studies [19, 20] and one of an empirical nature [22] have identified behaviors corresponding to IPA. Reynolds et al. developed a list based on the definition of IPA provided by the Australian Institute of Health and Welfare [19]. More recently, Stamatakis et al. suggested a preliminary list of common forms of IPA based on the compendium of physical activities [23], encompassing dimensions such as home activities, home repairs, gardening, walking, and biking, among others [20]. Finally, a recent study using a 24‐h written record in the university population [22] documented activities that responded to the dimensions described by Stamatakis et al. [20].

Advancing IPA research can inform public health policies and interventions aimed at increasing physical activity levels across different demographics, ultimately fostering healthier communities [10, 21]. IPA is inherently more accessible to diverse populations, including those who may not have the time, resources, or inclination to engage in formal exercise routines [5, 8, 9]. Integrating IPA into daily life through activities such as walking, taking stairs, and household chores can encourage a more active lifestyle to reduce the risk of chronic diseases, enhance mental health, and improve overall well‐being [14, 21, 24]. Consequently, in order to advance in this line of research, it is necessary to have a theoretical definition of the concept of IPA [15, 21] as well as to know the main empirical findings in IPA research. Therefore, the objectives of this review are: (1) To synthesize the definitions of the IPA used in the scientific literature; (2) To identify the behaviors that are part of the IPA; and (3) To synthesize the main empirical findings on IPA.

## 2 Method

### 2.1 Design

The reporting of this scoping review was conducted following the standards established by the PRISMA‐ScR statement [25]. The PRISMA checklist can be found in the Data S1. The study was registered on July 22, 2023 on the International Platform of Registered Protocols for Systematic Review and Meta‐analysis (INPLASY202370089).

### 2.2 Search Strategy

The search strategy followed the guidelines established by the Peer Review of Electronic Search Strategies (PRESS) [26]. EBSCOhost, Cochrane Library, Pubmed, ScienceDirect, Scopus, and Web of Science systematically searched the CINAHL databases.

The general search syntax was limited by title, abstract, and keywords. It was: (“incidental physical activity” OR “incidental physical activity of daily living” OR “incidental movement” OR “vigorous intermittent lifestyle physical activity” OR “VILPA” OR “physical activity of daily living”). In this way, the scope of the search was extended to all articles that explicitly dealt with IPA and studies that investigated this IPA under terms such as “incidental movement” or as part of the physical activity of daily living. The search looked at studies in English published up to July 2023 and then conducted a second update search up to February 29, 2024. The review was not limited by publication date, as the interest was to capture the evolution of the IPA concept. Gray literature was not included, as the focus was on published definitions, behaviors, and findings on IPA that have been peer‐reviewed.

### 2.3 Study Selection and Inclusion Criteria

All articles that met the search phrase were considered. Then, only those articles that met the following inclusion criteria were selected: (1) *Population*: children and adolescents, adults, older people, with or without health conditions; (2) *Study objectives*: articles whose main objective was the study of IPA or those that reported the measurement of such activity. In addition, we considered studies that investigated IPA by using the term “incidental movement” to refer to behaviors such as walking, stair climbing, active transport, home, occupational, and leisure‐time activities. Also, studies were incorporated under the term “physical activity of daily living” that included a set of behaviors that are part of the IPA, such as walking, climbing stairs, active transportation, home, occupational, and leisure activities; (3) *Variables*: IPA level, incidental movement, or physical activities of daily living; and (4) *Study design*: studies classified as instrumental research, empirical research (i.e., experimental, quasi‐experimental, single‐case, non‐experimental, qualitative, and mixed studies), and methodological research [27].

Articles were excluded when: (1) the study objective or outcome measure was physical exercise or structured physical activity (e.g., trekking or group classes such as yoga, dance, or fitness); (2) the study objective or outcome measure was incidental movements not identified as behaviors that are part of the IPA, such as eye movements or muscle spasms; (3) the study objective or outcome measure included physical activities of daily living without specifying the type of activities or behaviors performed, or recreational physical activities, such as recreational sports, hiking, dancing, and outdoor games, or was considered a categorical indicator of functional ability or disability; (4) were published as conference abstracts, posters, dissertations, theses, commentaries, study protocols, in non‐scientific journals, or systematic or literature reviews; and (5) the language was different from English or Spanish.

### 2.4 Data Extraction

After eliminating the duplicate records obtained from the databases through the Zotero bibliographic manager, the selection process was carried out using the Rayyan platform [28, 29]. The identified articles were independently reviewed by two reviewers (DR‐M and RZ‐L), and any discrepancies were discussed with a third reviewer (IC) until agreement was reached.

Data extraction in this scoping review was performed in two stages. First, two review authors (DR‐M and RZ‐L) selected records that met the inclusion criteria by reading the title and abstract of the articles. Second, when decisions could not be made solely based on the title and abstract, full‐text documents (DR‐M and RZ‐L) were retrieved. The inter‐reviewer agreement was analyzed using Cohen of the Kappa index during the study selection process [30]. The level of agreement among reviewers during study selection in the first stage was 82.0% (κ = 0.62; 95% CI: 0.52–0.72), indicating substantial agreement according to the classification of Cohen [30]. In the second stage, it was 88.6% (κ = 0.75; 95% CI: 0.66–0.84), indicating substantial to excellent agreement.

### 2.5 Data Synthesis Strategy

**FIGURE 1:**
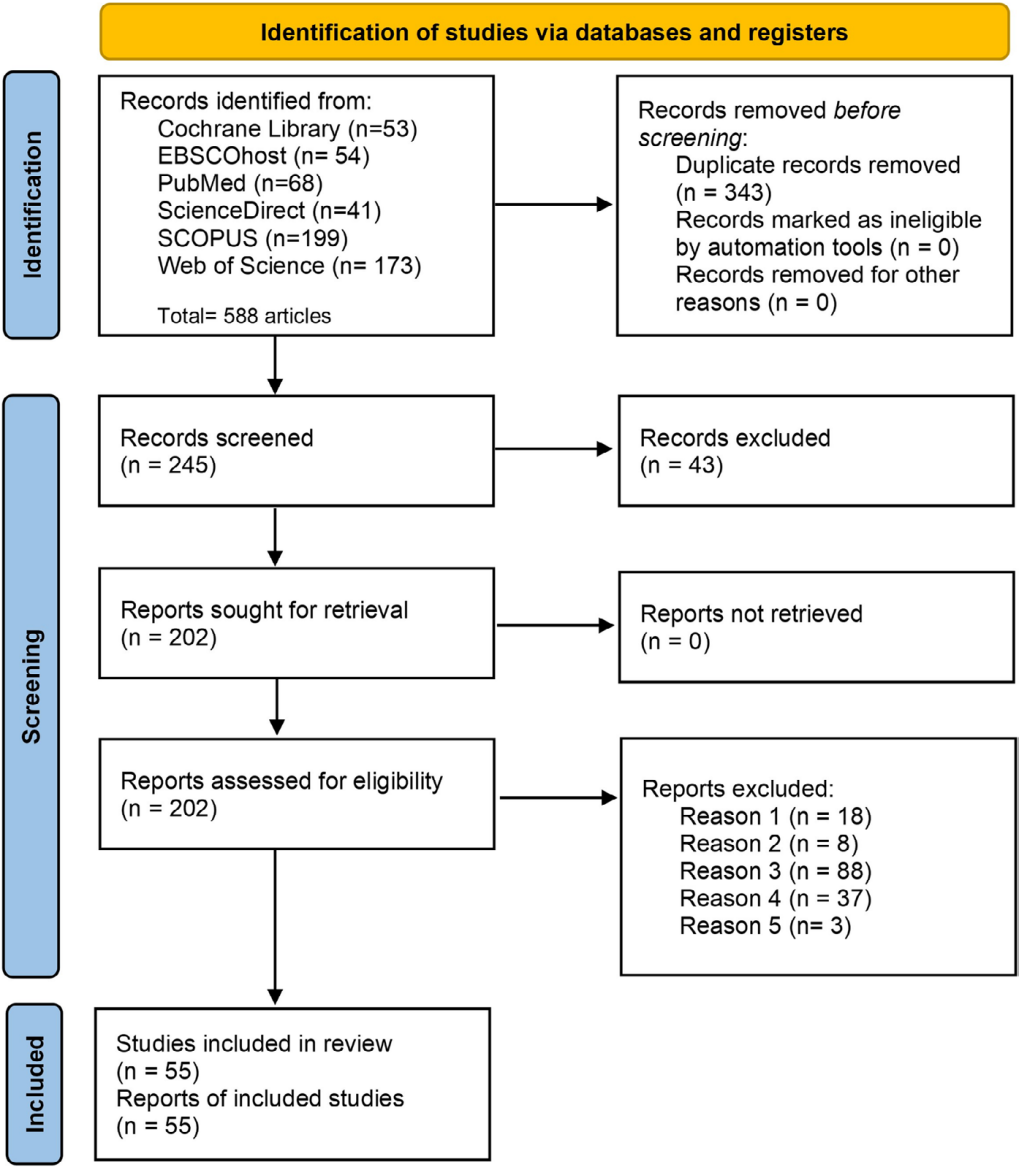
PRISMA statement study selection flowchart [31].

**FIGURE 2:**
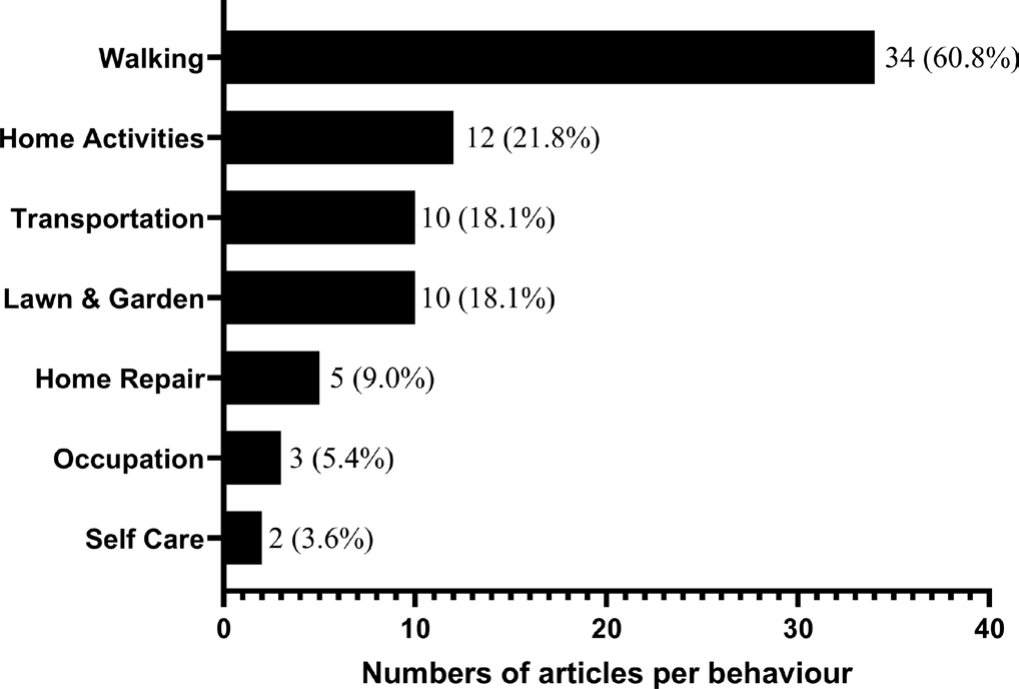
Frequency of articles by behavior categories according to main activities in the compendium of physical activities.

We summarized the evidence from the included studies and presented relevant information in tables and figures. The PRISMA flowchart represents the stratification of the selected studies (Figure 1). Table 1 provides a summary of the general characteristics of the included studies. Table 2 presents the conceptual definition of IPA used by the authors. To do this, the FITT principle (Frequency, Intensity, Time or Duration, and Type or Mode of Physical Exercise) established by the American College of Sports Medicine was used [89]. Given the characteristics of the IPA, the frequency domain of the FITT principle was not considered in this context. Table 3 summarizes the behaviors and instruments used to measure IPA. Figure 2 shows the frequency of behaviors reported in the IPA measurement by item. Behaviors reported in the IPA measurement were classified according to the 2024 Adult Compendium of Physical Activities [90]. Table 4 summarizes the main findings on IPA reported in the included articles.

**TABLE 1:** General characteristics of the included articles.

Reference	Country	Design	Population	Final sample	Mean age	Male/female/other %
Ahmadi et al. [32]**	United Kingdom	Prospective cohort	Non‐athletic population or population that performs physical exercise without cardiovascular diseases	23 292	61.8 ± 7.6	61/39
Alatorre‐Cruz et al. [33]	Mexico	Cross‐sectional	Older adults aged 60 years and older	36	60–77	—
Andrews et al. [34]	Australia	Cross‐sectional	People 65 years and older without neurodegenerative disease or severe psychiatric or neurological conditions	127	65–88	33/67
Assemi et al. [35]	Australia	Descriptive	Undergraduate engineering students at the University of Queensland	142	—	62/38
Beavis & Moodie [36]	Australia	Cross‐sectional	General population	29 840	0–80+	—
Bellettiere et al. [37]	United States	Quasi‐experimental	Pedestrians use stairs/escalators at the airport	1091	18–66+	33/77
Benítez‐Flores et al. [38]	Brazil	Randomized controlled	College students	30	19–35	50/50
Benítez‐Flores et al. [39]	Brazil	Randomized crossover	Healthy adults are physically active but not athletic	10	20–40	70/30
Bull et al. [40]	United States	Randomized controlled trial	Adult primary care patients	203	49.0 ± 6.4	26/74
Candido et al. [41]	Australia	Cross‐sectional	Office workers	20	—	—
Chudyk et al. [42]	Canada	Cross‐sectional	Older adults 65 years and older	151	70–79	34/66
Conroy et al. [43]	United States	Cross‐sectional	Undergraduate students	201	19.2	38/72
Coronini‐Cronberg et al. [44]	England	Cross‐sectional	Older adults 60 years and older	16 911	60–70+	46/54
D'Alonzo & Cortese [45]	Costa Rican/ United States	Cross‐sectional	Healthy Costa Rican Women	19	12–19	−/100
D'Alonzo & Sharma [46]	United States	Photovoice	Women	8	35–55	−/100
Engelen et al. [47]	Australia	Cross‐sectional	University of Sydney students and staff	—	—	—
Eves et al. [48]	United Kingdom	Quasi‐experimental	Passengers at the train station	—	—	—
Gilson et al. [49]	United Kingdom/ Australia/Spain	Quasi‐experimental	White‐collar university employees	179	41.3 ± 10.1	21/79
Harding et al. [50]	United States	Quasi‐experimental	Persons aged 60 or older diagnosed with cancer	41	70.1 ± 4.4	44/56
Khalil et al. [51]*	Jordan	Cross‐sectional	People with multiple sclerosis	50	36.7 ± 10.0	22/78
Kim et al. [52]	South Korea	Descriptive	Korean adult general population	1703	19–64	38/62
Koch et al. [11]	Germany	Cross‐sectional	Adolescents from a mental health institute without mental disorders, cardiovascular disorders, or chronic endocrine or immunological diseases	113	12–17	—
Macdonald et al. [53]	Australia	Cross‐sectional	Schoolchildren	34	—	59/41
Mark & Janssen [54]	United States	Cross‐sectional	General population	1165	8–17	—
Marshall et al. [55]	Australia	Quasi‐experimental	Hospital staff	40	—	22/78
McCormack et al. [56]	Australia	Cross‐sectional	General population	84	18–65	54/46
McGann et al. [57]	Australia	Mixed methods	Employees working in the government sector	90	—	—
McGuire & Ross [58]	Canada	Cross‐sectional	Inactive men and women with abdominal obesity	126	35–69	33/67
Merom et al. [59]	Australia	Randomized controlled trial	Inactive older adults	126	65–74	33/67
Munari et al. [60]	Brazil	Cross‐sectional	Subjects with a clinical diagnosis of chronic obstructive pulmonary disease who are clinically stable	131	40–80	28/72
Norrish et al. [61]	Australia	Cross‐sectional	Primary school children	64	10.5 ± 0.5	56/44
Oliver & Kemps [18]	Australia	Cross‐sectional	University student population and the community in general	103	17–68	31/69
Osborne et al. [62]	Australia	Randomized controlled	Recreationally trained males	8	26.5 ± 1.8	100/—
Pan et al. [63]	United States	Cross‐sectional	Patients needed to undergo a bilateral nephrectomy	9	33–71	66/34
Panisset & Galea [64]**	Australia	Retrospective cohort	People with multiple sclerosis	151	48.2 ± 11.3	87/11/1
Perry et al. [65]	Australia	Cross‐sectional	Older adults with osteoarthritis knee	19	55–81	47/53
Puig‐Ribera et al. [66]	Spain	Quasi‐experimental	University employees	190	42	35/65
Raisi et al. [67]**	United Kingdom	Prospective cohort	People who used stairs	442 027	37–73	46/54
Ross & McGuire [68]	Canada	Cross‐sectional	Inactive men and women with abdominal obesity	135	35–65	32/68
Ruff et al. [69]	United States	Cross‐sectional	Employees	1348	18–65+	45/55
Sanchez‐Lopez et al. [12]	Mexico	Cross‐sectional	Healthy older people	97	66.8 ± 4.3	34/66
Scharff et al. [70]	United States	Cross‐sectional	Women	611	18–75	—/100
Stamatakis et al. [14]	United Kingdom	Prospective cohort	Nonexercising adults	25 241	40–69	44/56
Stamatakis et al. [24]**	United Kingdom	Prospective cohort	Nonexercising adults	22 398	62.0 ± 7.6	45/55
Straiton et al. [71]	Australia	Cross‐sectional	Older adults with severe aortic stenosis	10	82.3 ± 7.0	47/53
Thogersen‐Ntoumani et al. [72]	Australia	Focus group interview	Physically inactive middle‐aged and older adult	78	35–76	76/23/1
Tonello et al. [13]	Brazil	Cross‐sectional	Young, non‐menopausal, overweight but healthy women, free of pathological conditions and medications, full‐time service workers	21	34.5 ± 6.4	—/100
Tudor‐Locke et al. [73]*	United States	Cross‐sectional	General population	3744	20+	48/52
Tudor‐Locke et al. [74]	United States	Cross‐sectional	General young population	54	20–36	37/63
Vancampfort et al. [75]	Uganda	Cross‐sectional	Outpatients with psychosis	50	33.5 ± 14.3	56/44
Vancampfort et al. [76]	Uganda	Cross‐sectional	Patients with depression	50	29.0 ± 14.2	70/30
Wallmann‐Sperlich et al. [22]	Germany	Cross‐sectional	University students and office workers	23	—	—
Wu et al. [77]	United Kingdom	Cross‐sectional	Healthy people who used stairs	451 699	40–69	45/55
Zheng et al. [78]**	United States	Cross‐sectional	Persons with multiple sclerosis	201	22–77	22/78
Ziviani et al. [79]	Australia	Cross‐sectional	Primary school children	164	9.1 ± 2.0	46/54

*Note:* ±, It represented the mean age's standard deviation (SD). +, The “plus” symbol after an age, such as 80+, indicates that the sample includes people aged 80 and older. *Articles were added to the systematic review because they were cited in the included articles. **Articles added in the update.

**TABLE 2:** Incidental physical activity definition.

Reference	Concept used	Definition	Intensity	Time	Type	Definition based on
Ahmadi et al. [32]**	MV‐ILPA	Short sessions of less than 10 min are carried out as part of activities of daily living, not related to participation in exercise or sport during leisure time or recreational walking more than once a week		✓	✓	—
Alatorre‐Cruz et al. [33]	IPA	Unstructured daily activities, such as housekeeping, working, transportation, etc.			✓	Strath et al. [80]
Andrews et al. [34]	IPA/Incidental activity	—				—
Assemi et al. [35]	IPA	Physical activity accumulated through normal daily activities unassociated with exercise goals, such as walking for transport purposes			✓	Reynolds et al. [16] Ross & McGuire [68]
Beavis & Moodie [36]	IPA	—				—
Bellettiere et al. [37]	IPA	Activities that are incidental to the routine, everyday living tasks, such as stair use			✓	Lavizzo‐Mourey & McGinnis [81]
Benítez‐Flores et al. [38]	IPA/Incidental lifestyle	Work, hours of sleep, etc.			✓	—
Benítez‐Flores et al. [39]	IPA	—				—
Bull et al. [40]	PADL	—				—
Candido et al. [41]	IPA	—				—
Chudyk et al. [42]	IPA	—				—
Conroy et al. [43]	IPA	Unintentional physical activity			✓	—
Coronini‐Cronberg et al. [44]	IPA	Physical activity is a byproduct of an activity with a different primary purpose (i.e., active transport, such as walking, cycling, and use of public transport)			✓	—
D'Alonzo & Cortese [45]	IPA	Household chores, walking to and from school or work, and spontaneous physical activity			✓	—
D'Alonzo & Sharma [46]	IPA/Incidental activity	Household, family care, and occupational pursuits			✓	—
Engelen et al. [47]	IPA	—				—
Eves et al. [48]	PADL	—				—
Gilson et al. [49]	Incidental walking	Step during the working task			✓	—
Harding et al. [50]	Incidental movement	< 40 steps/min	✓		✓	Tudor‐Locke et al. [82]
Khalil et al. [51]*	IPA/Incidental exercise	—				—
Kim et al. [52]	Incidental movement	1–19 steps/min	✓		✓	Tudor‐Locke et al. [82]
Koch et al. [11]	IPA/Incidental activity	Cleaning, climbing stairs, or running to the train			✓	Kanning et al. [83]
Macdonald et al. [53]	IPA Incidental movement	The child moved without being instructed; this was recorded as an incidental transition (e.g., collecting learning materials and moving to speak to the teacher/peers)			✓	—
Mark & Janssen [54]	Incidental movement	A movement that falls below an intensity is usually considered physical activity (e.g., fidgeting or walking around the home)	✓		✓	—
Marshall et al. [55]	IPA	—				—
McCormack et al. [56]	IPA/habitual IPA/incidental activity	Incidental activity is considered physical activity undertaken while performing other functions (usually a byproduct of the function rather than specifically planned physical activity). Examples include short walks to the shop, walks to school, or even to public transport			✓	Dunn et al. [84] Bauman et al. [85]
McGann et al. [57]	IPA	—				—
McGuire & Ross [58]	IPA	Physical activity that fails to meet the guidelines is characterized as IPA, which consists primarily of light physical activity and sporadic (e.g., bouts lasting < 10 min) moderate‐to‐vigorous physical activity that is accrued through activities of daily living. This includes, but is not limited to, activities such as dusting, vacuuming, and raking the lawn	✓	✓	✓	Tremblay et al. [6] Crouter et al. [86] Kozey et al. [87]
Merom et al. [59]	IPA	—				—
Munari et al. [60]	PADL	—				—
Norrish et al. [61]	IPA/Incidental breaks	—				—
Oliver & Kemps [18]	IPA	It consists of different sub‐behaviors, including domestic work (e.g., household chores, gardening), workplace activity (e.g., physical labour), transport (e.g., cycling to get from A to B), and leisure‐time activity (e.g., walking the dog)			✓	Tudor‐Locke et al. [7], Levine [5]
Osborne et al. [62]	IPA/Incidental activity	—				—
Pan et al. [63]	IPA	—				—
Panisset & Galea [64]**	IPA/Incidental activity	Purposeful activities include active transportation, dog walking, taking the kids to the park, gardening, and household maintenance			✓	—
Perry et al. [65]	IPA/Incidental activity	Casual day‐to‐day activities				—
Puig‐Ribera et al. [66]	Incidental movement	—				—
Raisi et al. [67]**	IPA	—				—
Ross & McGuire [68]	IPA	Non‐purposeful physical activity accrued through activities of daily living			✓	—
Ruff et al. [69]	IPA	—				—
Sanchez‐Lopez et al. [12]	IPA	Unlike structured physical activity, it results from unstructured daily activities, such as working, housekeeping, transportation, leisure, etc.			✓	Strath et al. [80]
Scharff et al. [70]	PADL	Activities such as house and yard work and occupational‐related physical activities			✓	—
Stamatakis et al. [14]	VILPA	Brief and sporadic (up to 1 or 2 min long) bouts of vigorous‐intensity physical activity are part of daily life, such as bursts of very fast walking while commuting to work, moving from place to place, or climbing stairs	✓	✓	✓	Rey‐Lopez et al. [88]
Stamatakis et al. [24]**	VILPA	Brief and sporadic (up to 1–2 min) bouts of vigorous physical activity during daily living, e.g., bursts of swift walking or stair climbing	✓	✓	✓	—
Straiton et al. [71]	IPA	Part of everyday living includes active transportation (e.g., walking), domestic chores, and non‐specific ambulation in domestic settings			✓	Alzahrani et al. [17]
Thogersen‐Ntoumani et al. [72]	VILPA	Brief, vigorous bouts of IPA lasting 1 or 2 min that are done during activities of daily living, such as carrying shopping bags, carrying children, or walking uphill	✓	✓	✓	Stamatakis et al. [21]
Tonello et al. [13]	IPA	Non‐purposeful physical activity accrued through activities of daily living			✓	Ross &McGuire [68]
Tudor‐Locke et al. [73]*	Incidental movement	Cadence of 1–19 steps/min	✓		✓	—
Tudor‐Locke et al. [74]	Incidental movement	Cadence of 1–19 steps/min	✓		✓	Tudor‐Locke et al. [73]
Vancampfort et al. [75]	IPA/Incidental activity	—				—
Vancampfort et al. [76]	IPA	—				—
Wallmann‐Sperlich et al. [22]	Incidental lifestyle physical activity	Activities are part of daily life and are not intended for recreational or health purposes without requiring optional time. Physical activities carried out at work, home, transportation, and leisure are distributed throughout waking hours. IPA represents the opposite of structured physical activity or exercise characterized by programmed, pre‐planned, and intentionally directed activities, e.g., visiting a gym, jogging, or other recreational activities, improving or maintaining physical fitness, performance, or health. Incidental lifestyle physical activities have an intensity greater than 6 MET or ≥ 14 on the Borg scale of 6–20	✓		✓	Stamatakis et al. [20]
Wu et al. [77]	IPA	—				—
Zheng et al. [78]**	Incidental movement	Cadence of 1–19 steps/min	✓		✓	Tudor‐Locke et al. [73]
Ziviani et al. [79]	IPA/Incidental activities	Walking to shops, the school or the park, climbing stairs in buildings, opening garage doors by hand, and getting up to change the television channel			✓	—

*Note: Intensity*, Activity intensity of the FITT principle of the ACSM. Time, The time or duration of the activity of the FITT principle of the ACSM. *Type*, Type of activity of the FITT principle of the ACSM. *Articles were added to the systematic review because they were cited in the included articles. **Articles added in the update.

Abbreviations: IPA, incidental physical activity; MV‐ILPA, Moderate‐to‐vigorous intermittent lifestyle physical activity; N/A, not applicable; PADL, physical activity in daily life; VILPA, vigorous intermittent lifestyle physical activity.

**TABLE 3:** Behaviors and instruments of incidental physical activity measurement.

Reference	Behaviors assessed	Compendium activity	Type instruments	Instrument names	Time of use	Device location
Ahmadi et al. [32]**	Household chores include utilitarian standing movements to iron a shirt, washing dishes, kitchen activities, cleaning rooms, ambulation, and heavy household outdoor chores (such as gardening). Walking to go to the supermarket or walking for occupational work. Active travel and high energy activities like actively playing with children	(5) Home activities (8) Lawn & garden (11) Occupation (16) Transportation (17) Walking	Accelerometer	Axivity AX3 triaxial accelerometer	7 days	Dominant wrist
Alatorre‐Cruz et al. [33]	Housework, yard work, caretaking and leisure activities, leisurely walking, standing, and moving	(5) Home activities (8) Lawn & garden (17) Walking	Questionnaire	Yale physical activity survey (YPAS, Spanish version)	N/A	N/A
Andrews et al. [34]	Walks for running errands (such as visiting the general practitioner or store), outdoor chores (such as house maintenance and gardening), and indoor chores (including housework and self‐care tasks)	(5) Home activities (6) Home repair (8) Lawn & garden (13) Self‐care (17) Walking	Questionnaire	Incidental and planned exercise questionnaire (IPEQ)	N/A	N/A
Assemi et al. [35]	Transport‐related walking	(16) Transportation	Cell phone	Mobil app ATLAS II	2 days	—
Beavis & Moodie [36]	Active transport (sum of travel‐related walking and cycling)	(16) Transportation	Survey	Victorian Integrated Survey of Travel and Activity 2007–08 (VISTA07–08)	N/A	N/A
Bellettiere et al. [37]	Stair use	(17) Walking	Interviews	—	N/A	N/A
Benítez‐Flores et al. [38]	—	—	Accelerometer	GT1M, Actigraph, Pensacola, FL, USA	7 days	Right hip
Benítez‐Flores et al. [39]	—	—	Accelerometer	GT3X, Actigraph, Pensacola, FL, USA	3 days	Non‐dominant wrist
Bull et al. [40]	Childcare, work in the home, home repair, and yard work	(5) Home activities (6) Home repair (8) Lawn & garden	Survey	—	N/A	N/A
Candido et al. [41]	Steps	(17) Walking	Accelerometer	Fitbit Charge 2	—	Wrist
Chudyk et al. [42]	Walk	(17) Walking	Travel diary	—	N/A	N/A
Conroy et al. [43]	Step	(17) Walking	Pedometer	OMRON HJ‐720ITC. Bannockburn, IL	—	—
Coronini‐Cronberg et al. [44]	Active transport, use of buses, and walking 3 or more times per week	(16) Transportation	Travel diary	—	N/A	N/A
D'Alonzo & Cortese [45]	Steps	(17) Walking	Pedometer	Digiwalker pedometer	3 days	Waist
D'Alonzo & Sharma [46]	(1) a typical day's activities, including household tasks, family/childcare, and occupational responsibilities; (2) examples of both habitual and IPA accrued throughout the day, including walking to work, to a neighbor's home, or grocery store	(5) Home activities (11) Occupation (16) Transportation (17) Walking	Photovoice	—	—	N/A
Engelen et al. [47]	Stair use	(17) Walking	Observations	—	N/A	N/A
Eves et al. [48]	Stair use	(17) Walking	Observations	—	N/A	N/A
Gilson et al. [49]	Step	(17) Walking	Pedometer	Yamax SW‐200	5 days	—
Harding et al. [50]	Step or walk at different intensities	(17) Walking	Accelerometer	ActivPAL3 micro monitor, PAL Technologies Ltd., Glasgow, UK	7 days	On to the midline of the mid‐right thigh.
Khalil et al. [51]*	Walks to get to places, and average daily time spent doing outdoor and indoor activities such as gardening and house cleaning	(5) Home activities (8) Lawn & garden (17) Walking	Questionnaire/accelerometer	Incidental and Planned Exercise Questionnaire (IPEQ)/ActiGraph; Pensacola, FL, USA	7 days	Waist
Kim et al. [52]	Step or walk at different intensities	(17) Walking	Accelerometer	GT3X, Actigraph, (Pensacola, FL, USA) for 7 days on the hip.	7 days	Hip
Koch et al. [11]	When the participants did not do physical exercise	—	Accelerometer/e‐diaries	Movisens Move‐II or Move‐III, movies GmbH/Motorola Moto G	7 days	Right hip
Macdonald et al. [53]	Collecting learning materials and moving to speak to the teacher/peers	(17) Walking	Observation	A modified version of the Observational System for Recording Physical Activity in Children‐Elementary School (OSRAC‐E)	—	—
Mark & Janssen [54]	Light activities performed while not participating in physical activity of at least a low intensity	—	Accelerometer	Actigraph 7124 uniaxial monitor, Ft. Walton Beach, FL, USA	7 days	Right hip
Marshall et al. [55]	Stair use	(17) Walking	Observational/device infrared motion‐sensing	—	1 day	—
McCormack et al. [56]	You are walking or bicycling on continuous trips that last 10 min or less and may have been taken, such as short trips that include errands such as walking to the stores to buy lunch or a newspaper, to public transportation, to school, to work, or to recreation—also, activities like climbing stairs or parking your vehicle far from your destination	(16) Transportation (17) Walking	Questionnaire	Western Australian Incidental Physical Activity Questionnaire (WAIPAQ).	N/A	N/A
McGann et al. [57]	Stair use, walking, standing	(17) Walking	Photographic/observations	—	N/A	N/A
McGuire & Ross [58]	—	—	Accelerometer	Actigraph GT3X, Pensacola, FL	7 days	Right hip
Merom et al. [59]	For the accelerometer, a low cut‐off point was considered that covered moderate‐intensity daily activities, such as grocery shopping, mopping, and vacuuming, as well as free‐time activities, such as playing with children and gardening. The questionnaire included activities such as walks, house maintenance and gardening, housework and activities inside the house, hours on your feet for housework, personal care, or caring for another person	(5) Home activities (6) Home repair (8) Lawn & garden (13) Self‐care (17) Walking	Accelerometer/ Questionnaire	GT1M, Actigraph, Pensacola, FL, USA/ Incidental and Planned Exercise Questionnaire (IPEQ)	7 days	Right hip
Munari et al. [60]	Steps; time spent seated, lying down, standing, walking; and in PADL with the metabolic equivalent of task (MET) ≥ 3	(17) Walking	Accelerometer	DynaPort MiniMod triaxial accelerometers (McRoberts BV, The Hague, the Netherlands)	2 days	—
Norrish et al. [61]	Steps were taken during recess and lunchtime each day	(17) Walking	Pedometer	Yamax Digi‐Walker SW200	10 days	—
Oliver & Kemps [18]	They removed the pedometer when working out at the gym, participating in a team sport, running, and walking to exercise. However, they were instructed to keep the pedometer on when walking to the shops, riding a bike to work, or engaging in activity for other transport‐related reasons	(16) Transportation (17) Walking	Pedometer	G‐Sensor Accelerometer Pedometer	7 days	—
Osborne et al. [62]	—	—	Accelerometer	GT9X ActiGraph, Pensacola, FL, USA	5 days	Non‐dominant wrist
Pan et al. [63]	—	—	Survey	—	—	—
Panisset & Galea [64]**	Active transportation, dog walking, taking the kids to the park, gardening, and household maintenance	(5) Home activities (6) Home repair (8) Lawn & garden (16) Transportation (17) Walking	Survey	—	N/A	N/A
Perry et al. [65]	—	—	Questionnaire	Incidental and Planned Activity Questionnaire	N/A	N/A
Puig‐Ribera et al. [66]	Step	(17) Walking	Pedometer	Yamax‐200	5 days	—
Raisi et al. [67]**	Stair use	(17) Walking	Survey	—	N/A	N/A
Ross & McGuire [68]	Duration and intensity of behaviors with counts/min equal to or greater than 100	—	Accelerometer	ActiGraph GT3X, Pensacola, FL.	7 days	Right hip
Ruff et al. [69]	Stair use	(17) Walking	Survey web	—	N/A	N/A
Sanchez‐Lopez et al. [12]	Housework, working, yard work, caretaking and leisure activities, leisurely walking, standing, and moving	(5) Home activities (8) Lawn & garden (11) Occupation (17) Walking	Questionnaire	Yale Physical Activity Survey (YPAS, Spanish version)	N/A	N/A
Scharff et al. [70]	Childcare, housework, home repairs, and yard work	(5) Home activities (6) Home repair (8) Lawn & garden	—	—	N/A	N/A
Stamatakis et al. [14]	Standing utilitarian movements (e.g., ironing a shirt, washing dishes), walking activities (e.g., gardening, active commuting, mopping floors), running/high energetic activities (e.g., active playing with children)	(5) Home activities (8) Lawn & garden (16) Transportation (17) Walking	Accelerometer/ Action cameras	Axivity AX3 triaxial accelerometer	7 days	Dominant wrist
Stamatakis et al. [24]**	—	—	Accelerometer	Axivity AX3 triaxial accelerometer	7 days	Dominant wrist
Straiton et al. [71]	Steps	(17) Walking	Accelerometer	Fitbit‐Flex (Fitbit Inc., San Francisco, CA, USA)	—	Wrist
Thogersen‐Ntoumani et al. [72]	—	—	—	—	—	—
Tonello et al. [13]	—	—	Accelerometer	GT1M, Actigraph, EE. UU	7 days	Right hip.
Tudor‐Locke et al. [73]*	Steps	(17) Walking	Accelerometer	Actigraph	7 days	—
Tudor‐Locke et al. [74]	Steps	(17) Walking	Accelerometer	ActiGraph GT1M Ft. (Walton Beach, Florida)	7 days	Hip
Vancampfort et al. [75]	Household chores	(5) Home Activities	Questionnaire	Luganda (or English) version of the Simple Physical Activity Questionnaire (SIMPAQ)	N/A	N/A
Vancampfort et al. [76]	—	—	Questionnaire	Luganda (or English) version of the Simple Physical Activity Questionnaire (SIMPAQ)	N/A	N/A
Wallmann‐Sperlich et al. [22]	All activities that were not structured physical activity as reported in the Bouchard activity diary and that recorded a heart rate reserve of 50% or more significant (Structured physical activity was defined as (i) exercise or performing sports, (ii) all activities in the categories conditioning exercise, running, and sports, (iii) for the activities mountain biking, dance workout/dance, aerobic, dancing, Nordic walking, swimming)	—	Optical heart rate sensor/questionnaire	Polar M600, Polar Electro Oy, Kempele, Finland/Modified version of Bouchard's activity diary.	—	Wrist
Wu et al. [77]	Frequency of stair climbing	(17) Walking	Questionnaire	—	N/A	N/A
Zheng et al. [78]**	Steps	(17) Walking	Accelerometer	ActiGraph GT3X+, Pensacola, FL, USA.	7 days	Non‐dominant hip
Ziviani et al. [79]	Walking to and from school	(16) Transportation	Questionnaire	—	N/A	N/A

*Note:* IPA: Incidental Physical Activity. Compendium activity: Main activities in the Compendium of Physical Activity. The numbers in parentheses correspond to the order within the 22 categories in the Compendium. N/A: Not applicable. *Articles were added to the systematic review because they were cited in the included articles. **Articles added in the update.

**TABLE 4:** Categories and findings about incidental physical activity.

Reference	Categories	Study aim	IPA findings
Ahmadi et al. [32]**	Health & wellness	Examined the associations of bouts of moderate‐to‐vigorous intermittent lifestyle physical activity (MV‐ILPA) and the proportion of vigorous activity contributing within these bouts with mortality and major adverse cardiovascular events (MACE)	Brief episodes of IPA, ranging from 1 to 3 min and less than 1 min, were linked to a reduced risk of mortality and major adverse cardiovascular events (MACE)
Alatorre‐Cruz et al. [33]	Health & wellness	Evaluated how two levels of IPA (high or low) affect working memory processing and how this, in turn, may affect morphosyntactic processing in older adults	The IPA promotes cognitive strategies to cope with working memory loads and morphosyntactic processing
Andrews et al. [34]	Health & wellness	Investigated the relationship between automatic habit strength and PA engagement in older people across three aspects of PA— planned exercise, walking for exercise, and IPA	Higher automaticity scores, which measure the intensity of physical activity habits, were related to more hours of IPA per week. Additionally, participants with a lower body mass index reported more hours per week of IPA
Assemi et al. [35]	Environmental factors	Evaluated the advantages of using a smartphone app for collecting accurate, fine‐grained, and objective data on people's transport‐related walking	IPA was higher in areas with high density and diversity of urban functions, including shops, restaurants, public services, recreational, residential, educational, and work areas. The presence of public transportation stops and utility services influenced IPA. Home, educational, and residential centers are key generators of walking trips. In this sense, IPA is more common in areas with greater pedestrian and bicycle connectivity, especially during daylight hours
Beavis & Moodie [36]	Environmental factors	Observed levels of IPA to travel in Melbourne, and after determining key correlates, used an economic model to estimate the potential long‐term health and health‐economic benefits of changes in active transport patterns	Users of public transport, pedestrians, and cyclists presented higher levels of IPA than those who used vehicles (being higher in pedestrians and cyclists). Furthermore, an inverse relationship was identified between the distance to the city center and the place of residence, with the level of IPA
Bellettiere et al. [37]	PA interventions and promotion	Evaluated the influence of sign prompts and participant factors, including past PA on stair ascent in an airport setting	Signs promoting the use of stairs at the airport were associated with increased stair climbing, regardless of participants' physical activity history or other covariates measured in the study
Benítez‐Flores et al. [38]	PA interventions and promotion	The combined effects of resistance and sprint training, with very short efforts (5 s), on aerobic and anaerobic performances and cardiometabolic health‐related parameters in young healthy adults were compared	After a 3‐week sprint and resistance training intervention, the concurrent group spent more time doing moderate‐intensity IPA than the sprint interval training group
Benítez‐Flores et al. [39]	PA interventions and promotion	Compared the acute responses to three time‐matched exercise regimens (sprint interval training, high‐intensity interval training, and continuous training)	No differences in IPA were observed after a session of sprint interval training, high‐intensity interval training, and continuous training, respectively
Bull et al. [40]	PA interventions and promotion	Compared the effectiveness of tailored, personalized and general health messages, and usual medical care in promoting leisure time physical activity and physical activities of daily living	Personalized and tailored health messages (e.g., based on exercise goals and preferences) increase physical activities of daily living, such as childcare, household activities, home maintenance, and gardening
Candido et al. [41]	PA interventions and promotion	Provided empirical evidence from studies conducted before and after relocation from contemporary open‐plan offices to activity‐based working spaces	After transitioning to a workspace based on the activity‐based work model, no changes were observed in workers' step counts or distance traveled, but their IPA increased. In addition, a reduction in sedentary time was evident
Chudyk et al. [42]	Environmental factors	To better understand the mobility habits of older adults with low income, (1) described the types of destinations older adults with low income most commonly travel to in 1 week, and (2) determined the association between the prevalence of neighborhood destinations and the number of transportation walking trips these individuals make (average per day)	Accessibility to destinations such as shopping centres within the neighborhood was positively associated with walking in older persons
Conroy et al. [43]	Health & wellness	Tested whether implicit attitudes can prospectively predict objectively assessed PA and specifically unintentional PA	Implicit attitudes positively predicted IPA
Coronini‐Cronberg et al. [44]	Environmental factors	Assessed the potential public health benefit of the National Bus Pass, introduced in 2006, which permits free local bus travel for older adults (≥ 60 years) in England	Seniors with a free bus pass were more likely to use active transportation and walk regularly (3 or more times per week) than those without the pass
D'Alonzo & Cortese [45]	Environmental factors	Compared habitual and IPA among native Costa Rican and Costa Rican American adolescent girls	Costa Rican teenage girls had significantly higher incident physical activity levels than their American peers
D'Alonzo & Sharma [46]	IPA features	Explored the influence of *marianismo* beliefs on participation in habitual and IPA among middle‐aged immigrant Hispanic women, using a community‐based participatory research approach and Photovoice methodology	Religious beliefs and socioeconomic pressures negatively influenced women's ability to participate in physical activities
Engelen et al. [47]	PA interventions and promotion	Assessed whether signage to promote stair use results in changes to the proportion of stair‐to‐elevator use	Motivational and directional signs did not increase the use of stairs in university buildings
Eves et al. [48]	PA interventions and promotion	Reported how the isovist (i.e., in the opposite direction to the traffic flow) of an intervention for stair climbing installed on the way out of the station influenced the response to the intervention	After an intervention with signs to promote the use of stairs, an increase in the use of stairs was observed only for the largest isovist. Additionally, stair use was more frequent during the morning rush hour and at higher levels of foot traffic
Gilson et al. [49]	PA interventions and promotion	Compared the impact two different walking strategies had on step counts and reported sitting times	After a workplace walking intervention, those who walked incidentally (taking advantage of the office's physical environment, walking in the workplace, and performing work tasks) showed decreased sedentary time
Harding et al. [50]	PA interventions and promotion	Examined changes in walking cadence among older cancer survivors who participated in an intervention to interrupt sedentary behaviors with LPA (standing and stepping)	Older adult cancer survivors increased their selected step cadence in a 16‐week intervention with reminders via a wrist device
Khalil et al. [51]*	IPA measurement	Assessed the convergent and criterion validity of the International Physical Activity Questionnaire (IPAQ) and the Incidental and Planned Exercise Questionnaire (IPEQ) in people with multiple sclerosis	The International Physical Activity Questionnaire (IPAQ) was more effective than the Incidental and Planned Physical Activity Questionnaire (IPEQ) in assessing IPA. However, both questionnaires overestimated physical activity intensities compared to accelerometry data
Kim et al. [52]	IPA features	Identified the stepping cadence patterns in Korean adults by using objectively measured accelerometer data to analyze the time spent in each cadence category by sex and age	Men spent more time on incidental movements than women. Furthermore, the average daily time in this category increases with age, being highest in the 50–59 age group
Koch et al. [11]	Health & wellness	An ambulatory assessment study applied accelerometry in combination with e‐diaries on smartphones in 134 individuals aged 12–17 years to investigate the specific influences of physical activity on mood in adolescents' everyday lives	Physical activity affects adolescents' moods in their daily lives, with variable effects depending on the type of activity. After engaging in IPA, teens reported feeling better and more energized
Macdonald et al. [53]	IPA features	Observed Year 1 children's physical activity and its contexts during school class time and identify opportunities to incorporate additional activity	First‐grade children engaged in IPA more frequently in small group activities than whole class activities
Mark & Janssen [54]	Health & wellness	Explored the independent effects of physical activity intensity and incidental movement on total and trunk adiposity	IPA was not negatively related to total or trunk fat in adults
Marshall et al. [55]	PA interventions and promotion	Evaluated whether a stair‐promoting signed intervention could increase the use of the stairs over the elevator in a healthcare facility	An intervention with motivational cues initially increased the use of stairs instead of the elevator. However, these effects were not maintained over time
McCormack et al. [56]	IPA measurement	Determined the test–retest reliability of items measuring habitual IPA, IPA behavior and sedentary behaviors	Adults can reliably remember the frequency of the usual IPA but not the duration
McGann et al. [57]	PA interventions and promotion	It highlighted the gap between management, design, and health‐promotion strategies in the workplace and sought to illustrate how the disciplines of architecture and health promotion can work together to reduce sedentary behavior and increase opportunities for IPA within the working day	In addition to the physical design of the workplace, managerial and cultural aspects influence the reasons that lead workers to carry out IPA
McGuire & Ross [58]	Health & wellness	Determined the association between objectively measured IPA and sedentary behavior with abdominal obesity in a sample of inactive men and women	IPA was not associated with abdominal obesity in inactive men and women
Merom et al. [59]	IPA measurement	Assessed the criterion validity and responsiveness of the Incidental and Planned Exercise Questionnaire (IPEQ) specifically developed for aging research	IPA questions from the Incidental and Planned Physical Activity Questionnaire (IPEQ) were correlated with light accelerometer intensity. Furthermore, it was observed that incidental activities are mainly of low intensity
Munari et al. [60]	Health & wellness	Identified Medical Research Council and COPD Assessment Test (mMRC) cutoff points to discriminate sedentary behavior and physical activity of daily living level of subjects with COPD and verify whether these cutoff points differentiate pulmonary function, health‐related quality of life (HRQOL), functional status, and mortality index in subjects with COPD	Those with Modified Medical Research Council (mMRC) scores greater than or equal to 2, and COPD Assessment Test greater than or equal to 16 and 20 exhibited lower levels of physical activity of daily living compared to those with mMRC < 2 and CAT < 16 and < 20
Norrish et al. [61]	IPA features	Examined the effect of school uniforms on the amount and perceived intensity of physical activity undertaken by 10‐year‐old children during play breaks at school	Girls, but not boys, were more active at recess, lunch, and in general when wearing their sports uniform than their winter uniform. The school uniform did not affect the children's physical activity levels
Oliver & Kemps [18]	Health & wellness	Investigated how motivation and implicit processes contribute to levels of IPA	Implicit attitudes, autonomous and controlled motivation, and attentional bias were associated with IPA
Osborne et al. [62]	PA interventions and promotion	Tested the hypothesis that heat acclimation training would detrimentally affect sleep variables and alter IPA compared to a thermoneutral training control condition	Participants adjusted their IPA behaviors to recover after a high training load, reflected in a decrease in light physical activity
Pan et al. [63]	Health & wellness	Evaluated the efficacy and utility of robotic single‐access bilateral nephrectomy (r‐SABN)	IPA increased in patients undergoing robotic single‐access bilateral nephrectomy (r‐SABN)
Panisset & Galea [64]**	Environmental factors	Examined the effects of lockdowns on exercise in Australians with multiple sclerosis according to disability levels, lockdown severity and health technology use	During the COVID‐19 pandemic lockdowns, a decrease in IPA was reported. Furthermore, in people with disabilities, this decrease was more significant for those with severe disabilities compared to those with mild disabilities
Perry et al. [65]	Health & wellness	Examined the effects of osteoarthritis and aging on muscle Na^+^‐K^+^ pump in 36 older adults (range 55–81 years), including 19 with osteoarthritis and 17 asymptomatic controls	Participants with knee osteoarthritis had higher levels of IPA than the asymptomatic control group
Puig‐Ribera et al. [66]	PA interventions and promotion	Assessed the short and mid‐term impacts of a workplace web‐based intervention on self‐reported sitting time, step counts and physical risk factors (waist circumference, body mass index, blood pressure) for chronic disease	An intervention for sedentary office workers showed positive changes by increasing IPA
Raisi et al. [67]**	Health & wellness	Investigated the association between flights of stairs used daily at home and all‐cause mortality and cause‐specific incidence and mortality	Stair use (> 15 times per day) was associated with a lower risk of all‐cause mortality, cancer, and chronic obstructive pulmonary disease
Ross & McGuire [68]	Health & wellness	Determined whether IPA, expressed as duration or intensity, was associated with cardiorespiratory fitness	A positive association was observed between the duration and intensity of IPA and cardiorespiratory capacity
Ruff et al. [69]	Health & wellness	Analyzed the associations between building characteristics, stair prompts, and stair use in large urban worksites	Instructions for climbing stairs increased its use. Living on higher floors and having a higher body mass index were negatively associated. Additionally, women used fewer stairs compared to men
Sanchez‐Lopez et al. [12]	Health & wellness	Evaluated the association of IPA level with cognitive functions and resting electroencephalogram (EEG) in healthy old participants	IPA was associated with better cognitive function and increased brain activity speed, as measured by the frequency of waves recorded in the electroencephalogram
Scharff et al. [70]	IPA features	Examined the rates and factors associated with physical activity in women of various ages	Family characteristics (e.g., having children) were associated with engaging in less structured and less intense physical activities of daily living among women aged 49 years or younger
Stamatakis et al. [14]	Health & wellness	Examined the association of Vigorous intermittent lifestyle physical activity (VILPA) with all‐cause, cardiovascular disease (CVD) and cancer mortality in 25 241 nonexercisers (mean age 61.8 years, 14 178 women/11063 men) in the UK Biobank	VILPA activity of up to 1–2 min is associated with lower risks of all‐cause mortality, cardiovascular disease mortality, and cancer mortality
Stamatakis et al. [24]**	Health & wellness	Evaluated the dose–response association of device‐measured daily vigorous intermittent lifestyle physical activity (VILPA) with incident cancer and estimated the minimal dose required for a risk reduction of 50% of the maximum reduction	Small amounts of VILPA were associated with a lower cancer risk overall
Straiton et al. [71]	Health & wellness	Explored the acceptability and feasibility of using wearable trackers to measure IPA in aortic stenosis patients before and after aortic valve replacement	After aortic valve replacement, the greatest improvements were seen in participants with the lowest incident physical activity
Thogersen‐Ntoumani et al. [72]	Environmental factors	Examined the barriers and enablers of VILPA among physically inactive adults using the Capability, Opportunity, Motivation, Behavior (COM‐B) model as a conceptual framework	The barriers to realizing the VILPA were identified, including physical limitations, perceptions of aging, need for knowledge, environmental restrictions, perceptions of effort and energy, and fear. On the other hand, there were facilitators, including convenience, reframing physical activity as purposeful movement, use of reminders, normalization of active choice, gamification, sense of achievement, health improvements, personally relevant rewards, the adjustment of identity, and the transition from effortful to deliberate deliberation habitual action
Tonello et al. [13]	Health & wellness	Investigated the relationships between objective PA levels, CRF, and cardiac autonomic indices in adult, regular non‐exercising female workers	In overweight adult women, IPA was associated with greater autonomic reactivation (e.g., better heart rate recovery)
Tudor‐Locke et al. [73]*	IPA features	Determined cadence patterns in free‐living adults, and in particular, time spent at increasing cadence increments, including 100 steps/min and beyond	Adults spend about 8.7 h a day with a cadence of 1 to 59 steps/min, 16 min a day with a cadence of 60–79 steps/min, 8 min with 80–99 steps/min, 5 min with 100–119 steps/min and 2 min with 120+ steps/min
Tudor‐Locke et al. [74]	IPA measurement	Evaluated the potential for using accelerometer‐determined ambulatory activity indicators (steps per day and cadence) to predict total energy expenditure (TEE) and physical activity energy expenditure (PAEE) derived from doubly labeled water (DLW)	The number of steps and cadence, measured by accelerometers, significantly predicted total energy expenditure and energy expenditure associated with physical activity, with greater precision in men than in women
Vancampfort et al. [75]	IPA measurement	Investigated the test–retest reliability of the 2‐min walk test (2MWT) and the concurrent validity with the 6‐min walk test (6MWT) in outpatients with psychosis	IPA explained some of the variance in the 2‐min walk test score
Vancampfort et al. [76]	IPA measurement	Investigated the test–retest reliability of the 2‐min walk test (2MWT) and its concurrent validity with the 6‐min walk test (6MWT) in Ugandan patients with depression. And explored practice effects and assessed the minimal detectable change (MDC) and clinical correlates with the 2MWT	Variability in IPA was associated with variability in the 2‐min walk test results
Wallmann‐Sperlich et al. [22]	IPA features	(1) assessed moderate‐to‐vigorous PA duration and distribution of intensity during waking hours ≥ 50% of heart rate reserve (HRR), (2) the type of PA was identified through daily assessment, (3) assigned these activities into structured and lifestyle incidental PA, and (4) Compared this information between students and office workers	There was no difference in the amount of IPA between students and office workers, but there was a difference in the type. Students included leisure and transportation; office workers included transportation, home, and leisure time
Wu et al. [77]	Health & wellness	Prospective evaluated the association of stair climbing with type 2 diabetes and assessed modifications by a genetic predisposition to type 2 diabetes	A greater number of stairs ascended was related to a lower risk of developing type 2 diabetes, especially in individuals with a low genetic predisposition to this disease
Ziviani et al. [79]	IPA features	Examined the extent to which Australian children walked to and from primary school and surveyed parents to identify factors influencing this behavior	Parents' perceptions of the importance of physical activity, parents' school transportation history, and the distance to school were important factors in children's participation in walking to and from school
Zheng et al. [78]**	Health & wellness	Compared peak 30‐min cadence, peak 1‐min cadence, and time spent in incremental cadence bands between persons with multiple sclerosis and healthy controls, and examined the associations between peak cadence and laboratory‐assessed physical function and walking performance	People with multiple sclerosis accumulated more IPA (1–19 steps/min) than those without the disease

*Note:* *Articles were added to the systematic review because they were cited in the included articles. **Articles added in the update.

Abbreviations: IPA, incidental physical activity; PA, physical activity; VILPA, vigorous intermittent lifestyle physical activity.

## 3 Results

### 3.1 Literature Review

Figure 1 presents the flowchart for systematic reviews proposed by the PRISMA statement [31]. A total of 588 potential items were identified. Subsequently, after the exclusion of duplicates in the databases, selection and eligibility criteria were applied, and 48 articles were included. In addition, seven articles that met the selection and eligibility criteria were included. Of these, five articles were identified in February following an update to the systematic search [24, 32, 64, 67, 78], and two were identified from findings in the citation of included articles [51, 73]. Thus, a total of 55 articles for narrative synthesis were included in this review (Figure 1).

### 3.2 General Characteristics of the Included Articles

The 55 articles reviewed were published between 1999 and 2024, and 37 were published in the last 10 years. Most of the studies were conducted in the Americas, with a total of 22 articles, and in Oceania, with 18 articles. Within the Americas, most of the research focused on North America, specifically the United States (13 articles), Canada (three articles), and Mexico (two articles). In South America, the research was limited to Brazil, with a total of four articles. All the studies conducted in Oceania were conducted exclusively in Australia, with 18 articles.

Most of the studies had non‐experimental designs (*n* = 40), with cross‐sectional designs being the most frequent (34 articles). There were 12 articles with experimental designs, two with a qualitative approach and one with a mixed‐method design. Participants were from all age ranges. Eight articles did not provide information on the age of participants. In 33 articles, the majority of the sample was composed of women, and in four studies, the research was conducted exclusively with women.

### 3.3 Incidental Physical Activity Definition

The term IPA was used in 38 of the 55 articles included. Then, the included articles identified more terms to refer to IPA. These terms were: incidental activity or activities, incidental movement, incidental walking, incidental breaks, incidental exercise, habitual incidental physical activity, incidental lifestyle or incidental lifestyle physical activity, vigorous intermittent lifestyle physical activity (VILPA), moderate‐to‐vigorous intermittent lifestyle physical activity (MV‐ILPA), and physical activity of daily life (PDLA). Of the 10 terms identified to refer to incidental physical activity, the most frequently used were “incidental activity or activities” (nine articles), followed by “incidental movement” (eight articles). The term “incidental movement” was used as a substitute for “incidental physical activity” in seven of the eight articles. In contrast, one article used it with the term “incidental physical activity”. On the other hand, in the nine articles in which “incidental activity or activities” was used, this term was used in conjunction with “incidental physical activity”.

In 33 articles, the concept addressed was defined (Table 2). Of those, 20 provided definitions for “incidental physical activity”, seven for “incidental movement”, six for “incidental activity or activities”, three for “VILPA”, two for “incidental lifestyle or incidental lifestyle physical activity”, one for “incidental walking”, one for “habitual incidental physical activity”, one for “PADL” and one for “MV‐ILPA”. Following the FITT principle of the ACSM, 11 articles incorporated *Activity Intensity* into the definitions of IPA and the related concepts. Intensity measures included step cadence, METs, or the Borg scale, which reported intensities from light to vigorous as part of IPA. Step cadence was the most frequently reported measure of intensity (in five studies), where the intensity of IPA ranged from 1 to < 40 steps/min.

*The time or duration of the activity* was incorporated into the IPA definitions of five articles, and although they all used minutes, all the descriptions were heterogeneous. The *Type of activity* was incorporated into the definitions of 32 articles, reporting most frequently that IPA is activities that are part of daily life (13 articles), performed in free time (two articles), without structure or planning (six articles), unintentional (four articles), that are performed as a by‐product of activity with a different primary purpose (three articles), and that do not have as their purpose the performance of exercise, sport, or physical activity (six articles) or recreational or health purposes (two articles). In addition, according to the definitions provided and following the Compendium of Physical Activities classification of activities [90], the IPA was comprised of Home Activities (13 articles), Lawn & Gardening (five articles), Occupation (seven articles), Transportation (13 articles), and Walking (10 articles).

In 17 articles, the definitions were based on previous publications, mainly by Ross and McGuire (2011), Strath et al. (2013), Tudor‐Locke et al. (2011), and Tudor‐Locke et al. (2017), being cited in two papers each.

### 3.4 Behaviors and Instruments of Incidental Physical Activity Measurement

From the 55 articles reviewed, 45 informed the behaviors measured as part of the IPA. In 41 of these 45 articles, the behaviors described were classified within the 22 main physical activities of the Compendium of Physical Activities for Adults [90]. In four of the 45 articles [11, 22, 54, 68], the details on the type of behavior considered for the measurement of the IPA were excessively broad or general, which prevented its classification according to the Compendium of Physical Activities. The behaviors described in the 41 articles fell into seven of the 22 possible Compendium categories, which were Home Activities, Home Repair, Occupation, Lawn & Garden, Self Care, Transportation, and Walking (see Figure 2 for the frequency of articles by behavior categories according to main activities in the Compendium of Physical Activities).

Various instruments and methods were used to measure IPA, including accelerometers, pedometers, cell phones, heart rate monitors, observation, interviews, photography, questionnaires, surveys, and diaries. Two articles did not provide information on the measuring instrument. The accelerometer was the most used instrument, present in 21 articles. The time of use of the accelerometers reported by the articles ranged from two to 7 days, with the 7‐day record being the most used in 16 of the 21 articles. In addition, accelerometers were used on areas of the body, such as the wrist, hip/waist, and thigh, with the most frequent location being the hip/waist (mainly on the right side) in 10 articles and then in seven articles the wrist (three on the dominant wrist, two on the non‐dominant wrist, and in two cases no information was provided). Two articles did not report the number of days of use of the accelerometer, and two others did not specify the use location. After accelerometers, the most used instrument was the self‐report questionnaire in 12 articles, followed by the Incidental and Planned Exercise Questionnaire (IPEQ) in four articles. Two articles did not provide information on the self‐report questionnaire used.

Figure 2 displays the frequency of articles for each of the seven main categories of the Physical Activities Compendium, into which the behaviors described in 41 of the 45 included articles were classified. Walking was the most frequently used behavior for measuring IPA in 34 articles (60.8% of the 55 articles) and included behaviors such as steps, using stairs, or when walking was reported to perform activities. Home Activities included behaviors such as cooking, ironing a shirt, mopping the floor, childcare, or when was reported to be household or housework. Transportation included behaviors such as walking to work or school, transport‐related walking, or when it was reported to be active commuting. Lawn & Garden included behaviors reported as gardening or yard work. Home Repair included behaviors reported as house maintenance or repair. Occupation included behaviors reported as walking for occupational work or working. Self care included behaviors reported as personal or self‐care.

### 3.5 Findings About Incidental Physical Activity

The findings on IPA were grouped into five categories: environmental factors, health and wellness, IPA features, IPA measurement, and physical activity interventions and promotion.

In 21 out of 55 included articles, findings related to health and well‐being were presented. Most studies on IPA directly associate it with health, specifically regarding the absence of disease and the reduction of mortality. In five of these articles, we found an association centred on higher IPA and a lower risk of all‐cause mortality, cardiovascular disease, cancer, and a lower risk of type 2 diabetes [14, 24, 32, 67, 77]. IPA and body composition were studied in four articles, two of which found no association with total fat or abdominal obesity [54, 58]. On the other hand, two other studies showed contradictory results. In one, participants with a lower body mass index (BMI) reported more weekly hours of IPA [34], whereas, in another study, it was reported that living on higher floors and a higher BMI were negatively associated [69]. Two articles explored the relationship between IPA and psychological variables, concluding that both implicit attitudes (i.e., those that manifest themselves subconsciously or automatically) as well as motivation, both autonomous and controlled, were associated with the level of IPA [18, 43]. Two articles explored the relationship of IPA with cognitive processes, observing an association with better cognitive function and greater speed in brain activity as an improvement in the comprehension and production of the grammatical structure of a language in older people [12, 33].

In 7 out of 55 articles, they presented findings on IPA and its relationship with contextual or environmental factors. Mainly, they stated that IPA was more common in urban areas with high density and diversity of functions, such as shops, restaurants, residential, educational, and work, especially during the day [35, 42]. The presence of public transport stops and free bus passes encouraged active transport and regular walking [35, 36, 44]. An inverse relationship was also identified between distance to the city center and place of residence with the level of IPA [36].

In 6 out of 55 articles, findings related to the measurement of IPA were presented [51, 56, 59, 74, 75, 76]. The use of accelerometers to measure IPA was mainly reported. The Incidental and Planned Physical Activity Questionnaire (IPEQ) was also compared with the accelerometer measurement, and the relationship between the IPAs and the 2‐min walk test was analyzed. The findings indicate that the IPAQ and IPEQ questionnaires underestimated IPA intensities compared to accelerometer records. In self‐report recording, adults can reliably remember the frequency of IPA but not the duration. In addition, the IPA level was associated with the results of the 6‐min walk test.

In 13 out of 55 included articles, findings on interventions for promoting physical activity were presented. Six of these articles reported on the use of signs and messages to promote IPA, and four of them showed positive effects on IPA levels, mainly in the use of ladders and walking (Bellettiere et al., 2017; Bull et al., 1999; Eves et al., 2009; Harding et al., 2022), while two others showed no effects [47, 55]. Four other articles reported that workplace interventions, including modification of workspaces, had positive effects on IPA levels, and two of these studies also mentioned reducing sedentary time. Three other articles reported on the effects of exercise‐based interventions on IPA, with an increase in the [38], a decrease [62], and no effect [39], respectively.

## 4 Discussion

This review sought to synthesize the definitions of the IPA used in the scientific literature. We found 10 different names in the literature to refer to IPA, and a conceptual definition was provided in 33 (60.0%) of the included articles. These definitions described the intensity of the IPA from light to vigorous, mainly through the cadence of steps ranging from 1 to 40/min. IPA included activities performed as part of daily life without structure, planning, intention, purpose of exercise, sport, recreation, or health. IPA definitions cover a wide range of durations, from brief episodes of less than a minute to prolonged activities such as housework or walking to school or work. This heterogeneity in the duration of IPA reflects the diversity of activities that are part of daily life. Behaviors composing IPA were primarily measured through accelerometers. They included walking, transportation, self‐care, occupations, gardening, home repairs, and home activities, with walking being the most commonly studied. On the other hand, evidence on IPA suggests an association with a lower risk of all‐cause mortality, including cardiovascular disease, cancer, and type 2 diabetes. Factors such as gender, age, parental beliefs, and environmental factors influence the level of IPA. In addition, interventions based on signage and messages in the workplace can promote the level of IPA.

### 4.1 Definition of Incidental Physical Activity

There is a consensus that IPA is part of daily life that excludes recreational activities. Similarly, there is agreement that IPA is unstructured, unplanned, and unintentional and performed as a by‐product of an activity with a different primary purpose. These findings clarify that unstructured and unplanned activities, such as active commuting or occupational physical activity, are considered IPA if performed unintentionally and as a by‐product of the occupation or the need to commute. Likewise, IPA can encompass activities of varying intensity and duration, ranging from light to vigorous and from extended activities to brief episodes of less than 1 min, such as what the literature recently calls VILPA [21], as long as it is unintentional. It is important to mention that the diversity of terminology used in the literature to refer to IPAs can lead to confusion. For this reason, it is recommended to avoid the use of concepts such as “incidental movement”, which has been used in the scientific literature both to refer to IPA and also to describe involuntary body movements, such as gestures or tonic spasms, associated with pathological conditions [91, 92, 93].

Certain discrepancies arose when analyzing the behaviors considered within the IPA measurement and the definitions provided by the included articles. Although walking was the most frequently reported behavior in the IPA measurement, various other behaviors were also identified, such as self‐care activities such as showering or brushing teeth. However, the definitions provided by the included articles did not explicitly include these self‐care behaviors as part of IPA. This gap in the literature between the behaviors used to measure IPA and the definition used by the authors to describe IPA highlights the need for an integrative conceptual definition based on a literature review, which aims to understand the concept completely.

Based on everything mentioned above, the reported results, and the previous analysis, we propose a novel integrative definition of IPA (Table 5). This definition, developed from a recognized theoretical framework and an exhaustive systematic literature review, describes the characteristics and behaviors that constitute IPA. In addition to the seven behaviors in Figure 2, we have added other examples of behaviors from the Adult Compendium of Physical Activities that also meet the IPA characteristics. Our purpose is to improve understanding and unify the concept of IPA, thereby addressing the gaps and resolving the contradictions in the current literature concerning its definition to facilitate the study and measurement of IPA. This comprehensive definition of the IPA concept aligns with previous proposals, including those in the editorial article by Stamatakis et al. [20], the observational study by Wallmann–Sperlich et al. [22], and the systematic review by Reynolds et al. [19].

**TABLE 5:** Integrative synthesis of the concept of incidental physical activity.

Integrative definition of the IPA	*Concept* Unstructured, unplanned, and unintentional physical activities of daily living are performed as a by‐product of an activity with a different primary purpose. These activities can be done during free time or in places of occupation. These activities do not have specific fitness, sport or recreation objectives. Include light and vigorous intensities ranging from short sessions of less than 1 min to prolonged ones *Behaviors* They include behaviors related to home activities (e.g., childcare or vacuuming), home repair (e.g., painting or washing a car), self‐care (e.g., showering, brushing teeth, eating), lawn & gardening (e.g., mowing the lawn), miscellaneous (e.g., standing talking in person or vacation involving walking), occupation (e.g., carrying objects, getting around), transportation (e.g., walking or biking to get to or from work or school), and walking (e.g., walking the dog or using stairs)

*Note:* The behaviors described here should always be reviewed against the conceptual definition of IPA, as some behaviors, although not structured or planned, may vary in the degree of intention with which they are performed, which could exclude them from being considered IPA.

Abbreviation: IPA: incidental physical activity.

In the measurement of IPA, the accelerometer in the right hip was the most used instrument, with 7 days being the most frequent use time. In this regard, the data collection and processing criteria using accelerometers to assess physical activity suggest use for 7 days, 24 h a day, but located on the wrist, as better user compliance and longer usage times have been reported [94, 95]. In particular, the recommendation is to place the accelerometers on the non‐dominant wrist [94, 95]. Otherwise, movements with the upper extremities, such as drawing, writing, brushing teeth, and playing with mobile electronic devices, can be considered time spent on physical activity [94, 95].

Given the complexity and variety of behaviors associated with IPA and the varying intensities and duration with which it can be conducted, self‐report questionnaires or step counting for its assessment are not recommended. Our results indicated that self‐report questionnaires underestimated the intensity of IPA, and it was further found that adults can reliably recall the frequency but not the duration of such activity [51, 56]. This is consistent with evidence suggesting that self‐reported physical activity has a low correlation with objective measurement methods, such as accelerometers [96, 97].

### 4.2 Findings on Incidental Physical Activity

The findings of the present review support an association between higher levels of IPA and a lower risk of all‐cause mortality, including cardiovascular disease, cancer, and type 2 diabetes. These results are consistent with the ample evidence of the positive effects of physical activity on health [1, 4] as well as growing evidence of the relationship between physical activity of any duration and improved health outcomes [2, 3]. Evidence indicates that performing intermittent stair‐climbing sessions for at least 1 to 3 min 3 days a week for 6 weeks leads to significant improvements in cardiorespiratory fitness [98, 99, 100]. Moreover, improved cardiorespiratory fitness is a protective factor against cardiovascular disease, cancer, and all‐cause mortality [101]. However, the evidence regarding mortality risk and IPA is mainly on VILPA [14, 24, 32]. Therefore, further evidence of IPA of any intensity and duration and its effects on health and mortality risk is required.

Among the background of the IPA, the importance of the environment and access to different urban areas and services stands out. Interventions that modify the environment, either through signage or modifications in the workplace, have shown positive effects on the level of IPA [37, 40, 41, 49, 50, 66]. These strategies focus on creating active environments, facilitating access to physical activity, and monitoring its performance through various technological devices [102].

### 4.3 Strengths and Limitations

To our knowledge, this is the first attempt to delineate the IPA concept through a systematic literature review. To ensure its methodological quality, systematic review guidelines were followed, and the protocol was registered on an international platform of registered systematic review and meta‐analysis protocols. It is important to note that, in the conceptual analysis of the IPA, guidelines created by recognized institutions such as the American College of Sports Medicine [89] were used, as well as the recently published update of the compendium of physical activities [90].

However, this review is not without limitations. First, in the systematic search, only studies that explicitly mentioned IPA or similar terms, such as incidental movement or physical activities of daily living that corresponded to IPA, were included. Therefore, it is possible that studies that did not use these terms were left out, which could have limited the findings on IPA. Second, due to the heterogeneity in the study designs of the included articles, it was not possible to analyze the risk of bias in these studies.

### 4.4 Perspective

Our findings indicate that IPA mainly involves walking‐related activities. While this is probably correct, it is also largely because the IPA measurements were made by counting steps and using stairs, behaviors that are considered part of walking [90]. For this reason, we hope that in the future, the integrative definition of IPA provided in this review and the use of accelerometers on the wrist non‐dominant 24/7 enable researchers to objectively and accurately report the proportions to which behaviors, such as those related to occupation, personal care, or household activities, contribute to IPA. However, at present, wrist‐worn accelerometers cannot accurately track activities like bicycle commuting. Therefore, it is crucial to advance the development of a protocol and specific algorithm for accelerometers capable of processing the information of all the behaviors that constitute the IPA, including bicycle commuting. Furthermore, it is necessary to advance in developing and incorporating measurement and analysis techniques that complement the information recorded by accelerometers, allowing only the behaviors corresponding to IPA to be filtered. In this sense, we suggest that future research also include methodologies such as action cameras [14] or digital activity diaries to identify the degree of intentionality or planning in the IPA classification [35]. An example of this would be the incorporation of Ecological Momentary Assessment (EMA) [103], which would allow the recording of the type of behaviors that individuals perform while using accelerometers. In this sense, it is crucial to incorporate tools that allow large‐scale data processing, such as the use of machine learning. In addition, we suggest avoiding classifications based solely on step ranges or focused on specific contexts, such as occupational activity or active commuting, as this limits the understanding of the variability of the IPA in terms of intensities and durations.

On the other hand, future research should explore how interventions aimed at increasing structured physical activity or modifying the environment impact IPA levels. In this context, it is essential to gain a deeper understanding of the individual, contextual, and environmental factors—such as motivation, barriers, and facilitators—that influence IPA. Population studies in low—and middle‐income countries, considering diverse sociodemographic characteristics such as age, sex, race/ethnicity, and socioeconomic level, are necessary [104]. Furthermore, there is currently no explanatory model of the IPA that considers environmental and individual variables. This would allow for a better understanding of IPA, developing theory‐based IPA interventions, and accurately identifying individuals at risk for incidental physical inactivity based on psychological and/or contextual variables.

## 5 Conclusions

This scoping review provides a proposal for a comprehensive definition of IPA based on the synthesis of evidence, covering a wide variety of behaviors and describing the situations in which it occurs. IPA emerges as a crucial component of total physical activity and carries significant health benefits, including reducing the risk of mortality from various causes such as cardiovascular disease, cancer, and type 2 diabetes. However, progress is needed in their measurement systems, particularly in the ability of the instruments to capture the diversity of behaviors that constitute the IPA and the data processing methods obtained.

Progress in measuring IPA will allow for understanding and developing explanatory models for this activity, considering contextual and individual variables. This perspective is crucial for establishing a solid theoretical basis in IPA and addressing current challenges of physical inactivity and its potential impacts on public health worldwide.

## Conflicts of Interest

The authors declare no conflicts of interest.

## Supporting information


Data S1.

## Data Availability

The data that support the findings of this study are available from the corresponding author upon reasonable request.
